# Critical Questions Surrounding the Shot-Blasting Treatment of Titanium Dental Implants

**DOI:** 10.3390/ma18174120

**Published:** 2025-09-02

**Authors:** Javier Gil, Eugenio Velasco-Ortega, Loreto Monsalve-Guil, Jesús Moreno-Muñoz, José Luis Rondón-Romero, Nuno Matos-Garrido, Álvaro Jiménez-Guerra, Enrique Núñez-Márquez, Iván Ortiz-García

**Affiliations:** 1 Bioinspired Oral Biomaterials and Interfaces, Departament of Materials Science and Engineeering, Escola d’Enginyeria Barcelona Est, Universitat Politècnica de Catalunya, Av. Eduard Maristany 16, 08019 Barcelona, Spain; 2 Comprehensive Dentistry for Adults and Gerodontology, Master in Implant Dentistry, Faculty of Dentistry, University of Seville, 41009 Sevilla, Spain; lomonsalve@hotmail.es (L.M.-G.); je5us@hotmail.com (J.M.-M.); jolurr001@hotmail.com (J.L.R.-R.); nunogarrido@orallagos.pt (N.M.-G.); alopajanosas@hotmail.com (Á.J.-G.); enrique_aracena@hotmail.com (E.N.-M.); ivanortizgarcia1000@hotmail.com (I.O.-G.)

**Keywords:** grit blasting, roughness, dental implants, titanium, osseointegration, bacteria

## Abstract

This review addresses four controversial aspects of shot blasting in the surface treatment of titanium dental implants. Shot blasting, which involves the projection of abrasive particles onto the titanium surface, is widely used to achieve surface roughness that promotes osteoblastic activity and, consequently, high levels of osseointegration. The first issue examined is the effect of residual alumina particles that remain embedded in the titanium surface after blasting. It has been shown that these residues—typically not exceeding 8% of the surface—can actually enhance osseointegration and even exhibit mild bactericidal properties. The second issue concerns the use of titanium dioxide particles for blasting. Our findings indicate that due to its low abrasiveness, titanium dioxide produces minimal surface roughness and low surface energy, resulting in limited osteoblastic adhesion, inferior fatigue performance, and reduced osseointegration compared to alumina-blasted surfaces. The third topic focuses on the role of compressive residual stress induced by grit blasting. Residual stress contributes to increased surface hydrophilicity, enhancing osteoblast adhesion and mineralization, as evidenced by elevated alkaline phosphatase levels. Finally, the fourth issue involves the effect of acid etching following grit blasting. This treatment introduces microroughness superimposed on the macroroughness generated by grit blasting. In vivo studies demonstrate that grit blasting is the primary driver of osseointegration, while acid etching provides only a marginal improvement in bone–implant contact.

## 1. Are Alumina Residues on Titanium Surfaces Harmful?

Sand blasting, grit blasting, or shot blasting are widely used surface treatment methods to optimize the roughness of titanium dental implants in relation to their biological performance. Alumina (Al_2_O_3_) particles are the most commonly used abrasive medium, often followed by acid etching. High-pressure grit blasting not only increases surface roughness but also induces significant compressive residual stress. However, a portion of the alumina particles remains embedded in the titanium surface.

Surface cleanliness is considered critical for successful osseointegration, and some studies suggest that inorganic contaminants may compromise clinical outcomes. Despite this, the impact of residual alumina on implant performance has not been thoroughly investigated, and conflicting findings are reported in the literature [1,2,3]. To address this gap, both in vitro and in vivo analyses were conducted using three surface conditions. Control specimens were machined, and two groups of implants were treated with alumina grit blasting. In one of the blasted groups, approximately 8% of the surface retained alumina particles, while in the other group, a rigorous ultrasonic cleaning process was applied to remove all residues.

**Figure 1:**
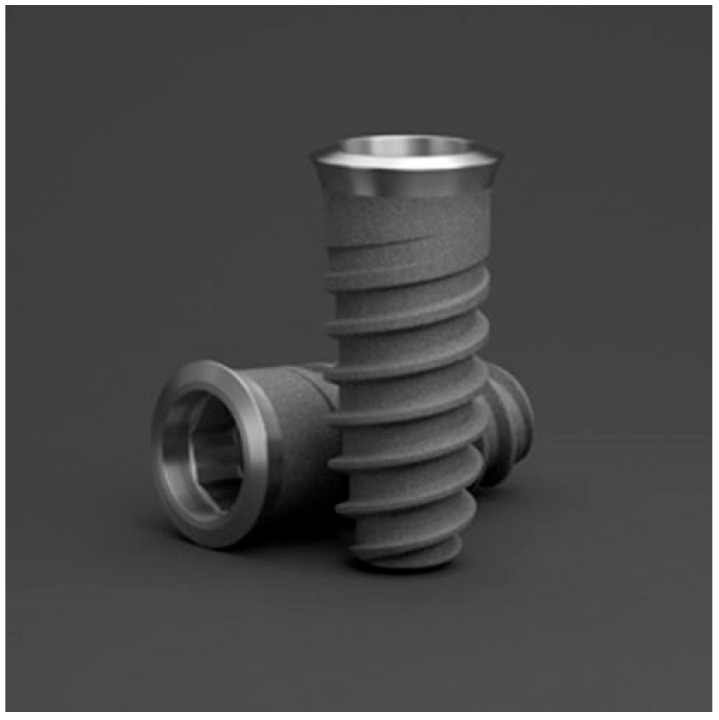
Essential dental implant (Klockner Medical Group, Madrid, Spain). The dental implants were 3.8 mm in diameter and 12.0 mm in length, with a pitch of 1.0 mm and a neck length of 1.5 mm. The dental implants were made with commercially pure titanium, grade 3.

The objective was to assess the effects of residual alumina on osseointegration and to compare the antibacterial properties of implants with and without alumina contamination. One study illustrating these effects involved three groups of implants with identical macro designs (Figure 1): (1) a control group with machined titanium surfaces, (2) a grit-blasted group using 200–300 µm alumina particles at 2.5 bar pressure, resulting in surfaces with approximately 8% residual alumina, and (3) a similarly grit-blasted group followed by ultrasonic cleaning to eliminate alumina residues. As is shown in Table 1, the surface roughness values of groups 2 and 3 were comparable, since identical blasting parameters were used.

**Figure 2:**
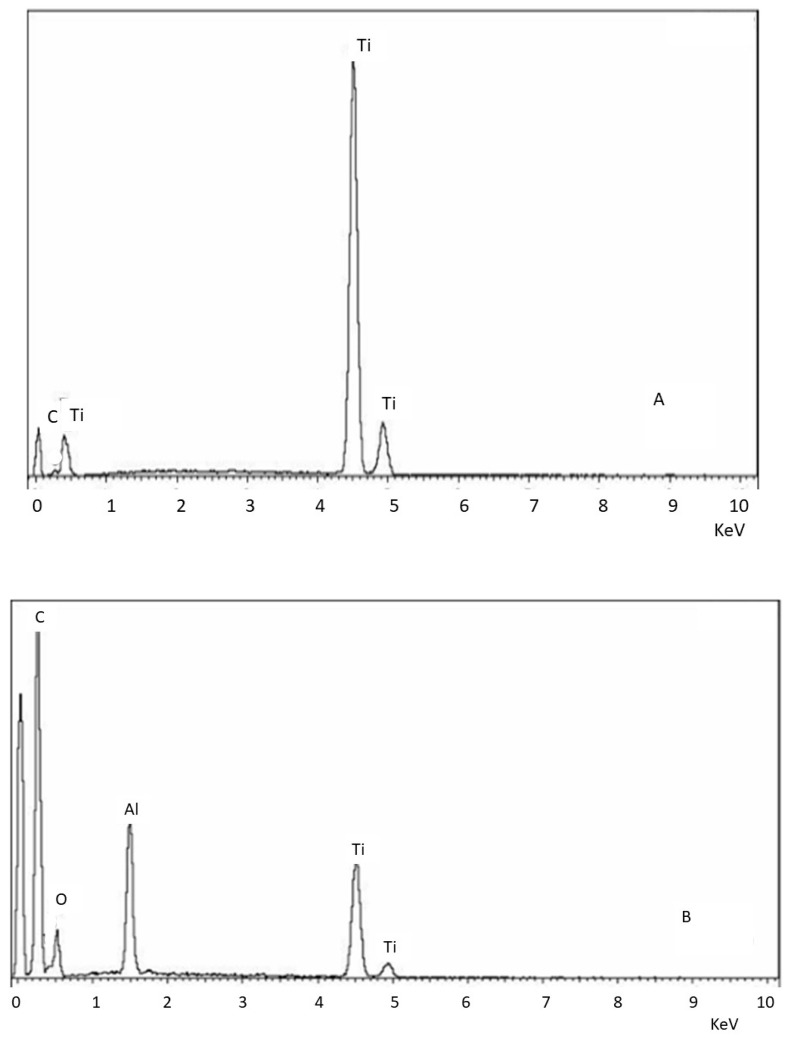

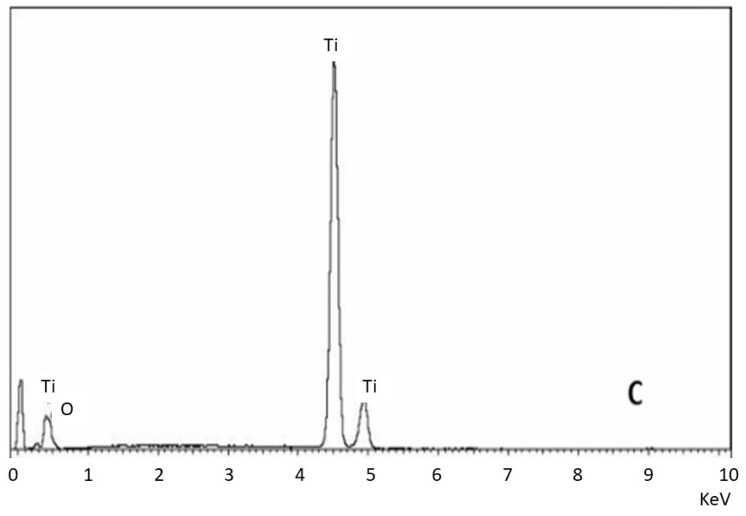
EDS microanalysis of the different surfaces. (**A**) Control. (**B**) Surface treated with sand blasting without cleaning. (**C**) Surface treated with sand blasting with severe ultrasounds cleaning.

Figure 2 shows the dispersive X-ray energy microanalysis, where it can be seen that the control and clean samples do not contain aluminum particles, while the sample that was not treated with ultrasonic cleaning shows the presence of aluminum oxide residues.

Table 1 presents the roughness results, showing that implants with and without residual alumina exhibit comparable roughness values. However, their physicochemical surface properties differ due to the presence of alumina residues. The same table includes contact-angle values corrected by the Denzel equation, total surface energy, and the contributions of its dispersive and polar components [4,5]. The data indicate that residual alumina increases the contact angle, reducing surface hydrophilicity, and decreases total surface energy. Interestingly, the polar component contributes significantly to the total surface energy, while the dispersive component remains low. This increased polarity is attributed to the alumina particles, which possess strong polar characteristics [6].

**Table 1:** Titanium roughness parameters: Sa, Sz and index area, contact angle corrected by Denzel equation CA’, and total surface energy with their dispersive and polar components. An asterisk (*) indicates a statistically significant difference compared with values without an asterisk. A double asterisk (**) indicates a statistically significant difference compared with values without an asterisk and those with a single asterisk. Statistically significant differences were considered at *p* < 0.05. (Adapted from Reference [6]).

Surface	Sa (µm)	Sm (µm)	Index Area	CA’ [^o^]	Surface Energy (mJ/m^2^)	Dispersive Component	Polar Component
Ctr	0.21 ± 0.02 *	0.34 ± 0.02 *	1.09 ± 0.01 *	66.3 ± 0.5 *	40.0 ± 3.5 *	24.8 ± 3.2 *	15.2 ± 4.0 *
Al_2_O_3_	2.35 ± 0.13 **	5.41 ± 0.21 **	1.18 ± 0.06 **	75.4 ± 0.5 **	28.2 ± 1.9 **	17.7 ± 1.1 **	10.5 ± 3.1 **
Clean	2.34 ± 0.25 **	5.67 ± 1.07 **	1.16 ± 0.04 **	66.8 ± 0.7 *	38.8 ± 2.5 *	26.8 ± 2.6 *	11.0 ± 3.4 **

Abrasive blasting increases surface roughness but reduces surface wettability and total surface energy, largely due to increased surface reactivity [7]. This effect is more pronounced in surfaces with residual alumina. Surface topography strongly influences cell interactions, including adhesion, orientation, migration, proliferation, and differentiation. These biological responses are governed not only by topography but also by physicochemical factors such as wettability, surface energy, and surface charge. Adhesive proteins like fibronectin—key in early bone development and osteoblast differentiation—prefer surfaces with higher wettability and surface energy [8,9,10,11].

For implant integration, a hydrophilic surface is desirable as it enhances cellular anchorage and vascularization in the peri-implant region, an area typically poor in vascular supply. Improved vascularization accelerates healing and reduces inflammation [12,13]. In this study, water contact angles were 75.4° for alumina-treated surfaces and 66.8° for cleaned ones. Although both are classified as hydrophilic (contact angle < 90°) [14], the lower angle of the cleaned surface suggests improved wettability.

The surface free energy of a solid comprises its polar and dispersive components. Greater non-polarity (higher dispersive character) typically reduces adhesive interactions, which depend on liquid–solid compatibility. In contrast, more polar surfaces enhance hydrophilic interactions and cell affinity [15]. Despite no statistically significant differences in the polar component between alumina and cleaned surfaces, both exhibited surface indentations and craters, which could reduce wettability and total energy compared to the control.

**Figure 3:**
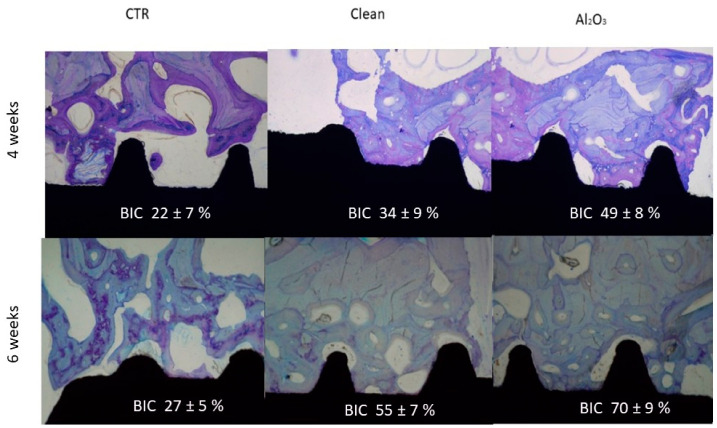
Histological images showing bone–implant contact at 4 and 6 weeks for the three tested surfaces (adapted from Reference [6]).

In vivo experiments in minipigs confirmed that implants with residual alumina exhibited successful osseointegration. Figure 3 shows histological cross-sections after 4 and 6 weeks of implantation, revealing significantly greater bone–implant contact in the alumina group.

**Figure 4:**
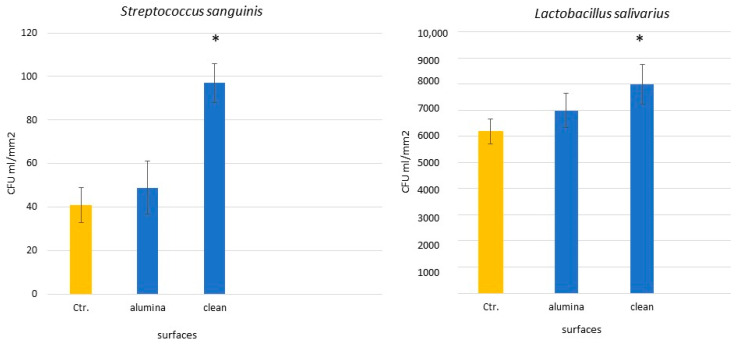
CFU quantification on titanium surfaces with and without alumina. Asterisks indicate statistically significant differences (*p* < 0.05). (Adapted from Reference [18]).

Bacterial adhesion to titanium surfaces involves several stages: bacterial migration, reversible and irreversible adhesion, specific interactions, and biofilm formation. Surface morphology and roughness, along with physicochemical factors like hydrophobicity and surface free energy, play crucial roles in these processes [16,17]. Residual alumina may also affect bacterial viability due to its oxidative properties. As is shown in Figure 4, bacterial counts for two strains related to peri-implantitis were lower on alumina-containing surfaces compared to cleaned ones.

Rougher surfaces tend to harbor more plaque, and while high surface energy promotes bacterial attachment, surface roughness remains the dominant factor. Our results confirm that sandblasted surfaces attract more bacterial adhesion than controls. However, surfaces with residual alumina showed reduced bacterial colonization compared to cleaned surfaces, likely due to lower surface energy and bacteriostatic properties of alumina [17,18].

While the presence of residual alumina is controversial, concerns about aluminum ion release into human tissues remain speculative and unsupported by direct evidence. Some studies suggest that aluminum ions can inhibit bone-marrow stromal cell differentiation and bone mineralization [2,19,20]. However, alumina (Al_2_O_3_) is bioinert, insoluble, and exhibits much lower ion release than Ti-6Al-4V alloy in simulated body fluids (SBFs) [21,22].

Many of these concerns stem from orthopedic applications, such as hip prostheses made entirely of alumina, which differ significantly from dental implants that contain only ~8% alumina on their surface and endure different mechanical loads [19,22].

Some authors suggest aluminum competes with calcium in the implant healing bed. For instance, Nimb et al. [23] reported aluminum inhibition of hydroxyapatite formation in dogs. In contrast, Quarles et al. [24] and Lau et al. [25] showed that aluminum ions could stimulate bone formation. Importantly, many such studies assess aluminum ions, not alumina itself—an important distinction, as metallic compounds often differ drastically in behavior from their ionic forms [1,26,27,28]. Feighan et al. [29] and Piattelli et al. [3] found that alumina-blasted implants supported active bone formation. Additionally, only trace amounts of aluminum were detected systemically when alumina was used [3]. Our findings agree: in vivo tests showed no toxic or inflammatory responses related to alumina, and implants with alumina residues displayed significantly higher bone–implant contact, likely due to enhanced wettability and surface energy [30,31].

Wennerberg et al. [32] observed similar trends in Ti-6Al-4V implants but also noted higher aluminum release than from Al_2_O_3_-treated implants. The amount of released aluminum from alumina was < 5 ppb over a month, indicating its chemical stability and negligible solubility [6,33,34,35,36]. Esposito et al. [37] reported that residual blasting particles were not responsible for implant fractures. Rather, tensile residual stresses from improper sandblasting caused such failures. Gil et al. [6] confirmed that alumina-treated implants did not show higher corrosion rates than cleaned ones, contrary to SiC-treated implants, which exhibited increased corrosion due to SiC oxidation into SiO_2_.

This aspect is controversial, but currently, the vast majority of dental implants are roughened by blasting them with alumina particles, with very good results. The release of Al^3+^ ions in dental implants treated with GTA 95 graphite furnace and 875 spectrophotometer ICP-MS has also been studied, and no significant presence has been detected in the physiological environment after several weeks due to the high stability of aluminum oxide [37,38,39,40,41]. Aluminum oxide is generally considered to be insoluble in water. This characteristic is attributed to the strong ionic bonds between aluminum and oxygen ions, which are difficult to break in aqueous environments. The energy of the Al_2_O_3_ network is high due to the small size of the aluminum ion and the strong electrostatic attraction between the ions, making it resistant to dissolution in water [42].

Aluminum oxide contains Al and O, with electropositive and highly electronegative elements, so the Al-O bond is ionic but not purely ionic. As a result, it has a covalent character, which can be used to describe the compound’s polarity [43,44]. Consequently, Al_2_O_3_ is negatively polar, though it is mostly ionic. It should be noted that alumina is very stable and considered bioinert, and there is no decomposition of the oxide, but what causes osteoblastic stimulation is the negative density due to the presence of three oxygen atoms, which favors the selective adsorption of proteins that promote bone cells [18,43,44]. Likewise, in vivo results show the good performance of implants with alumina residues. Thus, the acidic and oxidizing character of alumina allows it to have a bactericidal effect on some types of bacteria.

## 2. Is Shot Blasting with Titanium Oxide Suitable?

More than 75% of commercially available dental implants are treated with alumina particle blasting followed by acid etching. It is well established that the roughness generated by abrasive particle blasting plays a more critical role in successful osseointegration than acid etching. The latter contributes additional microroughness to the surface already modified by sandblasting [45,46]. However, some authors have raised concerns regarding the potential negative impact of residual alumina on the long-term osseointegration of dental implants. While previous studies have highlighted the benefits of alumina, this section focuses on the effects of sandblasting using titanium oxide (TiO_2_). One notable advantage of TiO_2_ is its chemical similarity to the implant’s titanium surface, thereby eliminating the risk of contamination from foreign compounds.

**Figure 5:**
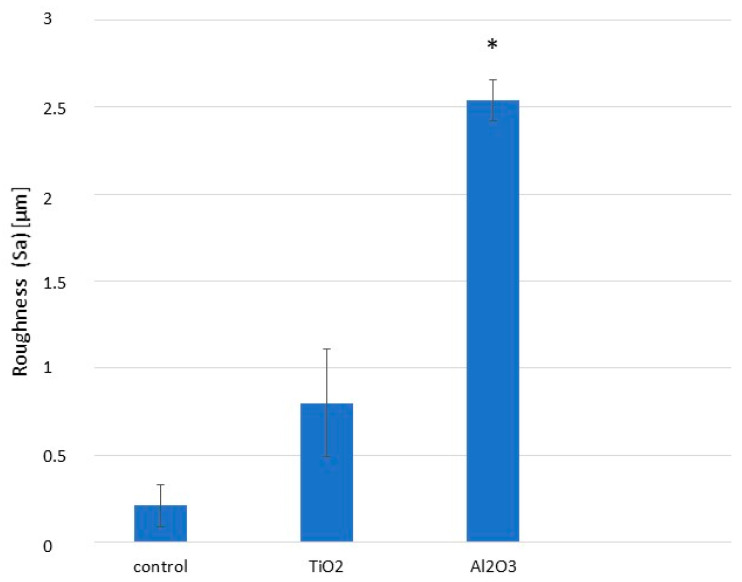
Surface roughness parameter (Sa) for machined, Al_2_O_3_-blasted, and TiO_2_-blasted titanium surfaces. Asterisk indicates statistically significant differences (*p* < 0.05). (Adapted from Reference [45]).

Bone-level implants were subjected to three different surface treatments and divided into three groups of 50 implants and discs each: (1) machining (control), (2) sandblasting with 600 µm TiO_2_ particles at a pressure of 0.25 MPa until roughness saturation, and (3) sandblasting with 600 µm Al_2_O_3_ particles at the same pressure and saturation criteria. Surface roughness is a key determinant of clinical success for dental implants. Surfaces treated with Al_2_O_3_ were significantly rougher than those treated with TiO_2_ or left machined, as shown in Figure 5.

Alumina demonstrated a higher abrasive capacity than titanium oxide, resulting in greater surface roughness (Figure 5). Surface roughness is crucial for implant success, and the roughness achieved with TiO_2_ was insufficient to meet osteoblast adhesion preferences, as previously reported by several authors [47,48]. A more abrasive particle is required to create an optimal surface for osteoblast activity; Al_2_O_3_ has been shown to provide roughness levels conducive to enhanced osteoblast attachment [49,50].

**Figure 6:**
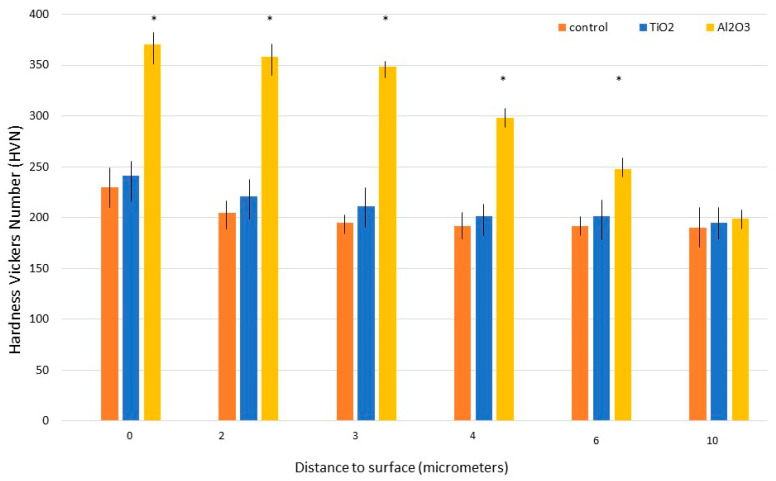
Microhardness variation with depth for machined, Al_2_O_3_-blasted, and TiO_2_-blasted titanium samples. Asterisks indicate statistically significant differences (*p* < 0.05). (Adapted from Reference [45]).

The cross-sectional microhardness distributions of the different treatments are shown in Figure 6. The maximum hardness measured for Al_2_O_3_-blasted samples was 370 ± 15 VHN, attributed to the material’s abrasive nature. Hardness values decreased with depth from the surface. TiO_2_-blasted samples exhibited a slight increase in hardness compared to the control, rising from 230 ± 14 VHN to 241 ± 11 VHN, owing to the impact of TiO_2_ particles. However, the relatively low abrasiveness of TiO_2_ resulted in only a modest improvement.

**Figure 7:**
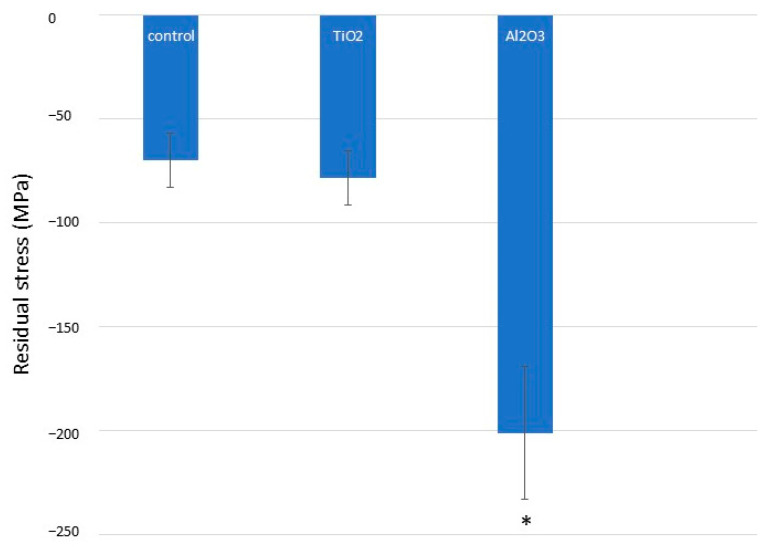
Compressive residual stresses for different titanium surface treatments. Asterisk indicates statistically significant differences (*p* < 0.05).

Residual stress measurements, obtained via X-ray diffraction using the Bragg–Bentano configuration, are presented in Figure 7. As expected, Al_2_O_3_ sandblasting induced significantly higher compressive residual stresses in commercially pure titanium compared to both control and TiO_2_-treated samples (*p* < 0.001). There were no statistically significant differences between the control and TiO_2_ groups. These findings correlate with the microhardness data, as higher hardness is typically associated with increased compressive residual stress.

**Figure 8:**
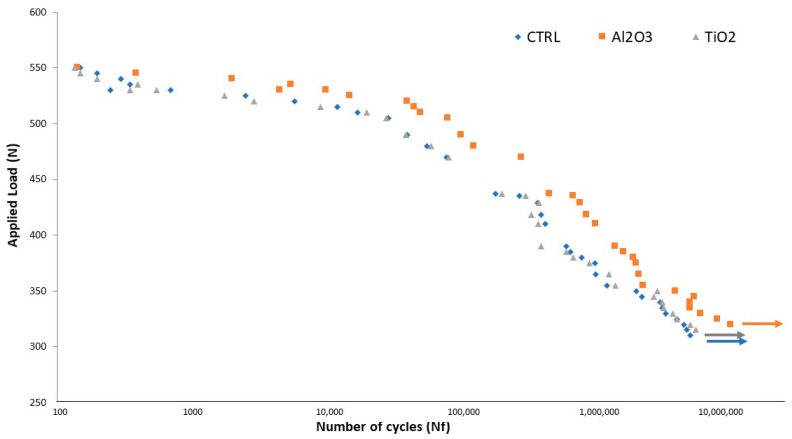
S–N curve for machined, TiO_2_-blasted, and Al_2_O_3_-blasted titanium implants (adapted from Reference [49]).

Fatigue testing results revealed that implants blasted with Al_2_O_3_ exhibited a longer fatigue life than those treated with TiO_2_ or that were left untreated (CTR), as illustrated in Figure 8. Differences between the Al_2_O_3_, TiO_2_, and CTR groups were statistically significant (*p* < 0.005). Under identical cyclic loading conditions, the number of cycles to fracture was consistently higher for Al_2_O_3_-treated implants.

The superior microhardness observed in Al_2_O_3_-blasted surfaces is attributed to its higher abrasive capacity, which in turn leads to increased compressive surface stress. This compressive state greatly enhances fatigue resistance. Fatigue cracks in dental implants typically initiate at the surface and propagate inward due to repeated mastication cycles. When the surface is in compression, crack nucleation at the surface is impeded, requiring cracks to initiate several micrometers beneath the surface instead. This compressive stress acts as a barrier to crack initiation, significantly improving fatigue behavior [45,51].

**Figure 9:**
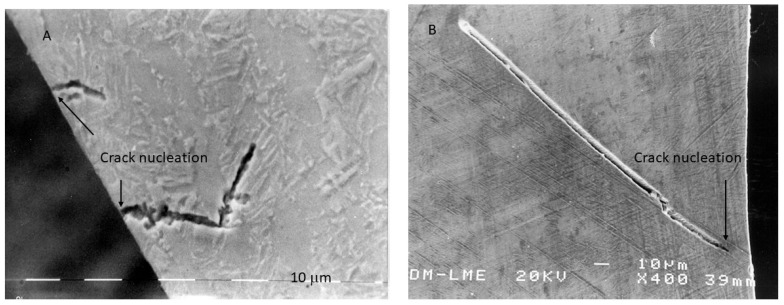
Fracture surface exhibiting crack nucleation from the surface for an implant that was subjected to TiO_2_ abrasive (**A**) and crack nucleation at a crack nucleation distance of 7 to 10 μm from the surface for the case of abrasion with Al_2_O_3_ particles (**B**). (Adapted from Reference [49]).

In addition to generating optimal surface roughness, Al_2_O_3_ blasting imparts a beneficial compressive residual stress state that hinders the formation of fatigue cracks, as shown in Figure 9. In TiO_2_-treated samples, cracks originate at the surface, whereas in Al_2_O_3_-treated samples, cracks initiate at a measurable depth from the surface. Since fatigue cracks generally form at stress concentrators under tensile conditions, a compressive residual stress field forces the crack initiation site into the subsurface region—approximately 9 µm below the surface in Al_2_O_3_-treated samples [45,50,51]. This substantially extends fatigue life and offers clinicians confidence in the long-term performance of the implants.

**Figure 10:**
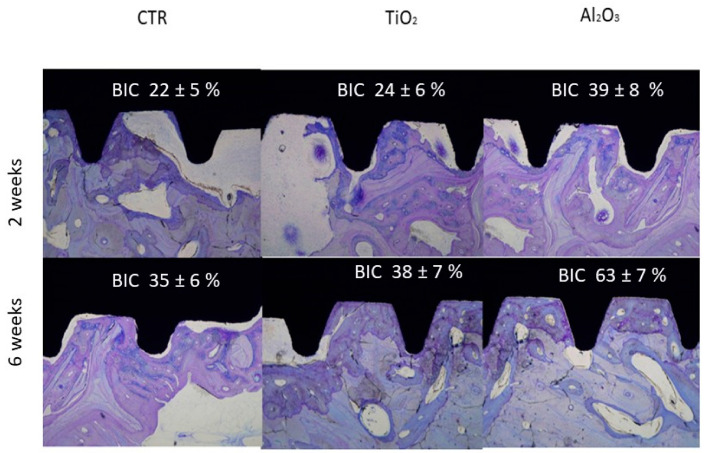
Histological sections of control (CTR), TiO_2_-blasted, and Al_2_O_3_-blasted implants at 2 and 6 weeks (adapted from References [45,49]).

In vivo testing is essential for assessing the biological response to dental implants. The objective of this study was to evaluate the osseointegration and mechanical properties of dental implants treated by sandblasting with titanium dioxide (TiO_2_) and alumina (Al_2_O_3_). The capacity of these surface-modified implants to promote new bone formation was investigated via in vivo implantation. Representative histological sections from each group—control (CTR), TiO_2_-blasted, and Al_2_O_3_-blasted groups—at 2- and 6-weeks post-implantation are presented in Figure 10.

Histological analysis showed increased bone–implant contact (BIC) from 2 to 6 weeks in all groups, indicating the ability of all surfaces to support partial or complete new bone formation. Importantly, the presence of residual abrasive particles (Al_2_O_3_ or TiO_2_) did not adversely affect osseointegration. At 6 weeks, implants treated with Al_2_O_3_ displayed superior bone integration, with extensive direct bone contact along the implant surface, compared to both TiO_2_-treated and control implants.

Bone–implant contact was further quantified through histomorphometric analysis, measuring the percentage of the implant surface in direct contact with bone. The results confirmed that Al_2_O_3_-treated implants had significantly higher BIC values at both 2 and 6 weeks, corroborating histological observations.

Two weeks may be considered a short amount of time in which to determine the osseointegration of dental implants. However, a considerable number of clinicians are currently placing dental implants and allowing loading after two weeks, which is considered early loading. There are also clinicians who, after placing the dental implant, allow loading from the outset, which is what they call immediate-loading implants [52,53,54]. In this case, the stability of the dental implant is achieved by compression of the coronal area of the dental implant with the cortical bone. In the case of early loading, bone formation helps with load transmission and prevents high compressive stress values in the cortex. The danger of overloading the cortical part of the bone is the possible lack of vascularization of the bone, causing bone necrosis [55].

Several studies have noted that residual alumina particles may enhance osteoblast adhesion, proliferation, and differentiation due to their negative surface charge, which promotes selective protein adsorption—especially fibronectin—thereby enhancing osteoblastic migration and surface colonization [46,47,48]. These findings, along with the widespread clinical use of Al_2_O_3_-blasted implants, suggest that any residual particles are not detrimental to osseointegration and may, in fact, be beneficial.

In contrast, TiO_2_ sandblasting typically produces surfaces with Sa values below 1 µm when using particles sized 12–300 µm. Although such microroughness can support osteoblastic activity, it is generally insufficient for optimal adhesion and proliferation. In a previous study using New Zealand rabbits, only ~50% BIC was achieved after 10 weeks with TiO_2_-blasted implants. Pull-out tests revealed a retention force of just 40 N after the same period [56].

Another comparative study assessed implants treated with Al_2_O_3_ + acid etching and TiO_2_ + acid etching, implanted in rabbit tibiae. The implant stability quotient (ISQ) and reverse torque measurements at 8 weeks showed no statistically significant differences between the two groups (*p* > 0.05) [57]. However, that study did not use standard Al_2_O_3_ for blasting, making the findings less comparable with those of our work.

In our study, comprehensive surface characterization—including roughness, microhardness, compressive residual stress, and fatigue resistance—was conducted. Al_2_O_3_-treated implants demonstrated superior biological and mechanical behavior. The increased surface roughness not only favored osteoblastic responses but also induced compressive residual stresses that significantly improved fatigue performance. This mechanical enhancement arises from the prevention of surface-crack initiation during chewing cycles.

The topography achieved through Al_2_O_3_ blasting was clearly more conducive to osseointegration than that produced by TiO_2_ due to its greater abrasiveness. This was reflected in the BIC values and the mechanical fatigue behavior. The induced compressive stress was confirmed via X-ray diffraction and microhardness testing, and contributed positively to implant longevity [58,59].

Attempts to use calcium phosphate particles for blasting were initially promising due to their potential bioactivity. However, their low abrasiveness and tendency to disintegrate on impact rendered them ineffective for roughening the titanium surface. Other efforts using calcium phosphate bioglasses showed excessive abrasiveness, causing deep surface cracks due to the sharp edges of glass particles, leading to premature implant failure and subsequent product recalls.

Titanium oxide lacks sufficient abrasiveness to create the optimal surface roughness required for enhanced osseointegration. Although it does not produce debris or contamination from compounds of a different chemical nature—thus offering advantages in chemical compatibility and reducing the risk of contamination—it is rarely used in dental implants to improve surface roughness and mechanical properties. However, titanium oxide passivation layers structured as nanospikes have been developed, exhibiting bactericidal properties without compromising the osseointegration of dental implants. Building on this nanotextured passivation-layer modification, ongoing research aims to develop permanent bactericidal implants that retain the full osseointegrative potential of conventional dental implants [60,61,62,63].

## 3. Do Surface Residual Stresses Contribute to Osseointegration?

As previously discussed, abrasive blasting creates plastic deformation in the metallic surface, producing macro- and micro-topographies that promote osteoblastic adhesion, proliferation, and differentiation. A by-product of this process is the development of residual compressive surface stress, which inhibits fatigue-crack formation and significantly extends implant lifespan. Beyond mechanical benefits, these residual stresses can also influence the physicochemical properties of the implant surface [64,65].

To investigate this, cp-Ti implants were divided into four groups:

S: Smooth titanium without residual stress
S+RS: Smooth titanium with residual stress
R: Roughened titanium without residual stress
R+RS: Roughened titanium with residual stress

For rough dental implants, 600 μm alumina particles were projected onto the titanium surface at a pressure of 2.5 bar and a distance of 150 mm between the surface and the projection gun. The diameter of the gun was 30 mm.

Contact-angle measurements were taken using the sessile drop method. Drops were generated with a micrometric syringe and were deposited over discs. A total of 3 μL of distilled water and 1 μL of formamide were deposited on each sample at 200 μL/min. Finally, the surface free energy was determined by applying the Owens, Wendt, Rabel, and Kaelble (OWRK) equation with wettability values obtained with distilled water and formamide, and the Wenzel equation for the correction of contact angles with the roughness [66].

Residual stresses were measured with a diffractometer incorporating a Bragg–Bentano configuration. The measurements were taken for the family of planes (213), which diffracts at 2θ = 139.5°. The elastic constants of Ti at the direction of this family of planes are EC = (E/1+ν)(213) = 90.3 (1.4) GPa. Eleven Ψ angles, zero and five positive- and five negative-angles were evaluated. The position of the peaks was adjusted with a pseudo-Voigt function using appropriate software (WinplotR, free access online, Compact version 2016), and then converted to interplanar distances (dΨ) using Bragg’s equation. Production of the d Ψ vs. sen2Ψ graphs and the calculation of the slope of the linear regression (A) were carried out with appropriate software (Origin, Microcal, version 5.3 code 137684 Amherst, MA, USA). The residual stress is as follows: σ = EC(1/d_0_)A, where d_0_ is the interplanar distance for Ψ = 0° [67,68].

To achieve a residual stress of approximately −200 MPa, as in the case of surfaces treated by abrasive blasting, compression stresses were applied to the titanium samples using an MTS Bionix servo-hydraulic machine, applying different compression loads. After each test, the residual stress was analyzed, and the one that most closely resembled the grit-blasting tests was the compression of 1050 MPa.

Table 2 presents the measured values for roughness, contact angle, and surface energy components.

**Figure 11:**
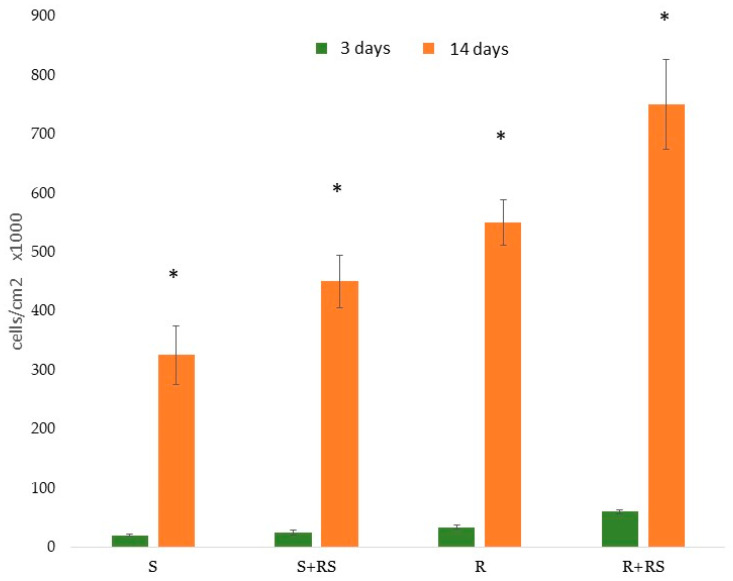
Proliferation of Saos-2 cells on the surfaces studied. After 3 days and 14 days of incubation. Asterisk indicates statistically significant differences (*p* < 0.05). (Adapted from Reference [69]).

**Figure 12:**
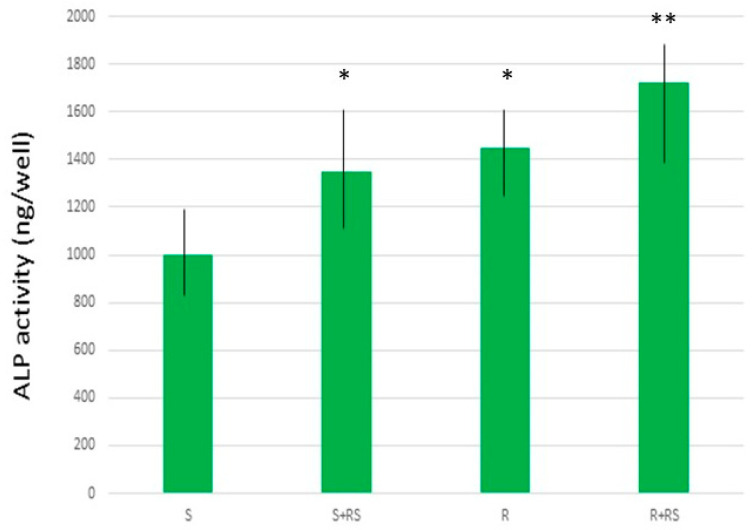
ALP production by Saos-2 cells on different surfaces. An asterisk (*) indicates a statistically significant difference compared with values without an asterisk. A double asterisk (**) indicates a statistically significant difference compared with values without an asterisk and those with a single asterisk. Statistically significant differences were considered at *p* < 0.05. (Adapted from Reference [69]).

The presence of compressive residual stress reduced the contact angle (i.e., increased hydrophilicity), especially in the roughened samples, and significantly improved both total surface energy and its polar component. Figure 11 and Figure 12 show that residual stress also enhanced osteoblastic cell proliferation and ALP (alkaline phosphatase) activity [68,69,70].

These findings confirm that while surface topography is the dominant factor for osseointegration, compressive residual stress has a synergistic effect—enhancing protein adsorption and supporting cell attachment and mineralization [71,72].

**Figure 13:**
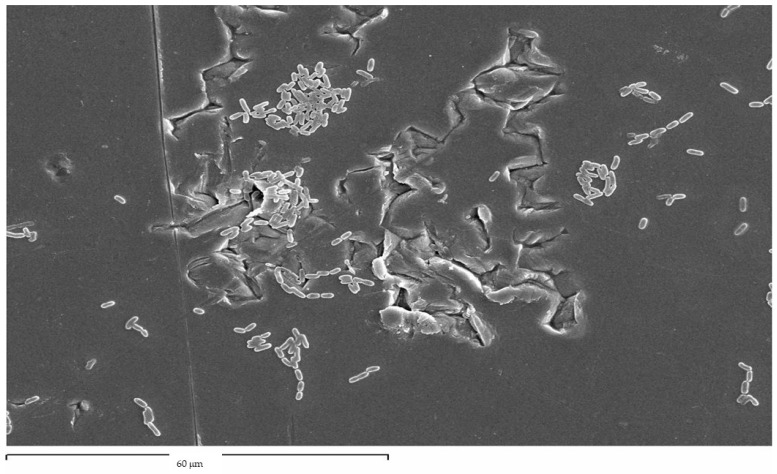
Streptococcus sanguinis colonized the grain boundaries of titanium dental implants.

As can be seen, compressive residual stress improves the behavior of osteoblastic cells but also, as we have seen above, compressive residual stress improves fatigue resistance because compression hinders crack nucleation on the surface and this fact delays initiation and therefore propagation, making the dental implant have a longer fatigue life. Regarding the subject of surface energy, we see that it affects the surface energy and also the polar component, favoring the adhesion, proliferation, and mineralization of osteoblasts, but this energy also favors bacterial colonization, as was observed in the studies of Rodriguez et al. [73]. In this study, it was observed that the bacteria grow preferentially at the grain boundaries, since these are the areas with the highest energy. This fact can be observed in Figure 13. Current research aims at obtaining titanium with compressive residual stress to favor osseointegration but modifying its surface with bactericidal or at least bacteriostatic elements to avoid the formation of biofilms that cause peri-implantitis.

The cause of the improvement in biological activity due to residual compressive stresses is not well understood, although various authors have been able to verify the influence of stresses on biological behavior [74,75,76]. One consideration is that compressive stress increases surface energy, and this fact favors interactions with the environment in order to lower this internal energy. This can be seen, for example, in the corrosion of metals, where the most stressed parts are the most favorable points for corrosion to occur. This has always been explained by the decrease in internal energy in accordance with the laws of thermodynamics. This behavior could be transposed to biological activity. In any case, a more in-depth study of the mechanisms that promote this increase in cellular and microbiologic behavior is necessary.

## 4. Does the Acid Etching After Shot Blasting Have Any Influence?

From this study, it can be concluded that the most important parameter of osteoblastic behavior on the surface is the topography, but the synergistic effect of compressive residual stress on the implant surface is also demonstrated. This osseointegration-promoting effect is due to the fact that stress reduces the contact angle and consequently affects the surface energy, especially the polar component. This improves the adsorption of proteins on the implant surface.

A prerequisite for the clinical success of titanium dental implants is the achievement of a strong and durable connection between the implant and the bone. It is well known that the micrometer surface roughness of dental implants is a very important factor in achieving clinically reliable bone fixation. In many brands of dental implants, shot blasting and subsequent acid etching is performed. With this question, we want to determine how each technique contributes to osseointegration [77,78,79]. Shot blasting with abrasive alumina particles produces a higher surface roughness than acid etching. What the acid etching produces is a micro-roughness within the macro-roughness produced by the blasting [80,81,82,83,84]. The question is whether this micro-roughness will improve osseointegration or whether there will be no significant difference to the macrorough surface, i.e., that which has only been treated by alumina sputtering.

Cp-Ti implants with four different surface treatments were studied and divided into four groups: machining (Ctr); acid etching (AEtch) in 0.35 M hydrofluoric acid for 15 s at room temperature; sandblasting (SBlast) with alumina particles (600 µm size) at a pressure of 0.25 MPa until roughness saturation is reached; and shot blasting with acid-etch treatment (SBlast+AEtch). Roughness and physicochemical properties can be observed in Table 3.

Both the SBlast and SBlast+AEtch surfaces had a significantly higher roughness than the AEtch and control samples. Although acid etching introduced some micro-roughness, it did not improve osseointegration beyond that achieved by sandblasting alone.

The SBlast+AEtch and SBlast surfaces exhibited significantly higher roughness values than both the AEtch and control (Ctr) groups. Similarly, AEtch surfaces were significantly rougher than those in the control group. However, no statistically significant differences were observed in surface roughness between the SBlast and SBlast+AEtch groups. This indicates that acid etching following grit blasting does not significantly alter the macro-roughness produced by blasting alone. It is important to note that the nanotopographic features generated are smaller than the lateral resolution limit of the measurement technique used.

**Figure 14:**
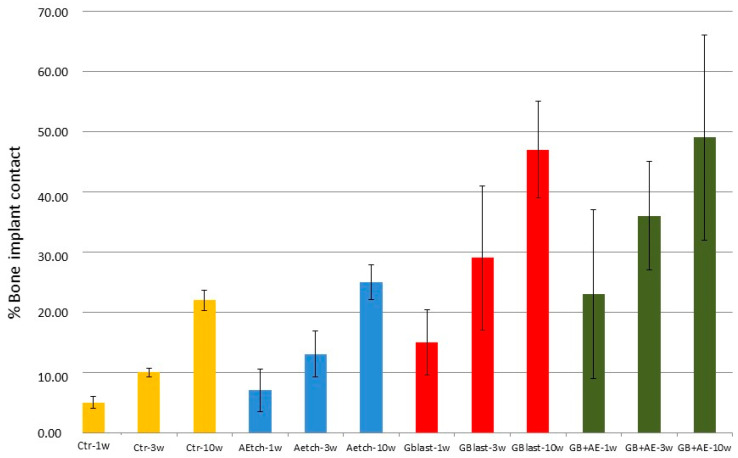
Osseointegration percentages for each surface treatment at various time points following implantation (adapted from Reference [86]).

Figure 14 presents the histomorphometric analysis of osseointegration across the different surface treatments. Control implants consistently exhibited lower percentages of bone in direct contact compared to both the SBlast and SBlast+AEtch groups, with statistically significant differences observed from the first week post-implantation (Fisher’s test, *p* < 0.05). This supports the conclusion that a high surface roughness (Ra ≈ 4 µm) promotes early and improved bone integration relative to machined surfaces (Ra ≈ 0.7 µm). In contrast, AEtch implants (Ra ≈ 1.5 µm) did not demonstrate a significant improvement in osseointegration compared to the control group at any time point up to 10 weeks post-implantation.

Both the SBlast and SBlast+AEtch groups demonstrated statistically significant improvements in bone–implant contact compared to surfaces in the AEtch and control groups. However, no significant differences were observed between the SBlast and SBlast+AEtch groups at any time point, indicating that acid etching following grit blasting does not further enhance osseointegration beyond that achieved by blasting alone. These findings suggest that accelerated osseointegration can be achieved with grit-blasted surfaces, with or without subsequent acid etching, as early as one-week post-implantation.

Surface roughness and topography are among the most influential surface characteristics in determining the biological performance of dental implants. Increased surface roughness correlates with enhanced osteoblastic cell adhesion. This effect was particularly evident in grit-blasted and grit-blasted plus acid-etched surfaces. The results strongly support the conclusion that the macro-roughness induced by grit blasting is the primary determinant of biological response, with acid etching playing a secondary role [84,85,86,87]. Although the impact of acid etching on osseointegration values is significantly lower than that of abrasive-particle projection, this treatment is used on practically all dental implants that undergo grit blasting. In addition to a certain improvement in osseointegration, acid treatment cleans the surface of any organic compounds that may have remained on the surface and stabilizes the titanium dioxide passivation layer, improving corrosion resistance and reducing ion release [87,88,89,90,91,92].

These conclusions were corroborated by in vivo experiments and histological evaluations.

## 5. Conclusions

This review highlights that residual alumina particles, even at concentrations of approximately 8% of the titanium dental-implant surface, may enhance bone formation and exhibit mild bactericidal properties. This effect could be related to the polar nature of titanium oxide and its acid–oxidative character, although further studies are needed to clarify the influence of the physicochemical surface properties on biological and microbiological activity. In contrast, titanium dioxide, while avoiding surface contamination and thus presenting a lower risk of toxicity, lacks sufficient abrasiveness to achieve the optimal roughness required for osteoblastic activity. Moreover, TiO_2_ blasting does not induce significant compressive residual stresses, which are crucial for improving fatigue resistance, resulting in osseointegration levels similar to those of untreated controls. Compressive residual stress generated by sandblasting was found to increase surface hydrophilicity, thereby enhancing osteoblast adhesion and mineralization, as well as contributing to long-term mechanical performance. The addition of acid etching to sandblasted surfaces led to a slight improvement in bone–implant contact, but the main contribution to osseointegration was attributed to the roughness generated by sandblasting. Acid etching, however, also improves other properties, such as the stability of the passive layer, thereby enhancing corrosion resistance. These findings should be considered by dental-implant manufacturers to optimize surface treatments and by researchers seeking to elucidate the underlying mechanisms responsible for these effects.

## Figures and Tables

**Table 2:** Roughness parameters: Sa, contact angle (CA), surface energy dispersive component (DC), polar component (PC), total surface energy (SFE), and residual stress (σ_residual_). An asterisk (*) indicates a statistically significant difference compared with values without an asterisk. A double asterisk (**) indicates a statistically significant difference compared with values without an asterisk and those with a single asterisk. In the same way, statistically significant differences compared with values without an asterisk, or with a single or double asterisk are represented by triple asterisks. Statistically significant differences were considered at *p* < 0.05. (Adapted from Reference [69]).

	Sa (µm)	CA (˚)	DC (mJ/m^2^)	PC (mJ/m^2^)	SFE (mJ/m^2^)	σ_residual_ (MPa)
S	0.21 ± 0.02 *	77 ± 5 *	24.8 ± 1.2 *	10.2 ± 2.0 *	35.0 ± 3.2 *	−10 ± 2 *
S+RS	0.24 ± 0.10 *	58 ± 3 **	27.2 ± 1.2 **	18.3 ± 1.8 **	45.5 ± 2.2 **	−189 ± 20 **
R	2.04 ± 0.15 **	69 ± 4 *	27.7 ± 1.3 **	12.5 ± 2.1 *	40.2 ± 1.2 **	−8 ± 3 *
R+RS	1.99 ± 0.18 **	53 ± 2 **	29.0 ± 2.2 **	20.4 ± 1.9 **	49.4 ± 1.8 **	−201 ± 12 **

**Table 3:** Roughness parameters Ra and Pc, contact angle, and total surface energy for the different types of cp-Ti implant surfaces. An asterisk (*) indicates a statistically significant difference compared with values without an asterisk with *p* < 0.05 (Adapted from Reference [84]).

Surface	R_a_ (μm)	P_c_ (cm^−1^)	CA’ [°]	Total Surface Free Energy
Ctr	0.33 ± 0.1	150.9 ± 69	66.3 ± 5	42.74 ± 1.54
AEtch	1.69 ± 0.1	198.3 ± 34	66.8 ± 7	49.52 ± 3.11 *
SBlast	4.74 ± 0.2	82.1 ± 10	75.4 ± 5 *	42.67 ± 1.18
SBlast+AEtch	4.23 ± 0.2	92.1 ± 13	82.1 ± 5 *	43.08 ± 1.96

## Data Availability

No new data were created or analyzed in this study. Data sharing is not applicable to this article.
